# The Temporal Expression Pattern of Alpha-Synuclein Modulates Olfactory Neurogenesis in Transgenic Mice

**DOI:** 10.1371/journal.pone.0126261

**Published:** 2015-05-11

**Authors:** Sebastian R. Schreglmann, Martin Regensburger, Edward Rockenstein, Eliezer Masliah, Wei Xiang, Jürgen Winkler, Beate Winner

**Affiliations:** 1 Department of Neurology, University Hospital Zurich, Zurich, Switzerland; 2 IZKF Junior Research Group III and BMBF Research Group Neuroscience, Interdisciplinary Center for Clinical Research, FAU Erlangen-Nürnberg, Nikolaus-Fiebiger Center for Molecular Medicine, Erlangen, Germany; 3 Department of Neurology, FAU Erlangen-Nürnberg, Erlangen, Germany; 4 Department of Neurosciences, University of California San Diego, La Jolla, California, CA, United States of America; 5 Department of Pathology, University of California San Diego, La Jolla, California, CA, United States of America; 6 Institute of Biochemistry, Emil-Fischer-Zentrum, FAU Erlangen-Nürnberg, Erlangen, Germany; 7 Department of Molecular Neurology, FAU Erlangen-Nürnberg, Erlangen, Germany; University of Wurzburg, GERMANY

## Abstract

**Background:**

Adult neurogenesis mirrors the brain´s endogenous capacity to generate new neurons throughout life. In the subventricular zone/ olfactory bulb system adult neurogenesis is linked to physiological olfactory function and has been shown to be impaired in murine models of neuronal alpha-Synuclein overexpression. We analyzed the degree and temporo-spatial dynamics of adult olfactory bulb neurogenesis in transgenic mice expressing human wild-type alpha-Synuclein (WTS) under the murine Thy1 (mThy1) promoter, a model known to have a particularly high tg expression associated with impaired olfaction.

**Results:**

Survival of newly generated neurons (NeuN-positive) in the olfactory bulb was unchanged in mThy1 transgenic animals. Due to decreased dopaminergic differentiation a reduction in new dopaminergic neurons within the olfactory bulb glomerular layer was present. This is in contrast to our previously published data on transgenic animals that express WTS under the control of the human platelet-derived growth factor β (PDGF) promoter, that display a widespread decrease in survival of newly generated neurons in regions of adult neurogenesis, resulting in a much more pronounced neurogenesis deficit. Temporal and quantitative expression analysis using immunofluorescence co-localization analysis and Western blots revealed that in comparison to PDGF transgenic animals, in mThy1 transgenic animals WTS is expressed from later stages of neuronal maturation only but at significantly higher levels both in the olfactory bulb and cortex.

**Conclusions:**

The dissociation between higher absolute expression levels of alpha-Synuclein but less severe impact on adult olfactory neurogenesis in mThy1 transgenic mice highlights the importance of temporal expression characteristics of alpha-Synuclein on the maturation of newborn neurons.

## Introduction

Dementia with Lewy bodies (DLB), Parkinson`s disease (PD), and multisystem atrophy (MSA) share the neuropathological hallmark of aggregation of alpha-Synuclein (a-syn) in various cell populations throughout the brain. The mechanisms leading to the accumulation and aggregation of this protein are still a matter of ongoing studies [1].

In the adult brain, neurogenesis is present throughout life in two distinct regions: the hippocampal dentate gyrus and the subventricular zone (SVZ) [2]. All mammals share this phenomenon to variable degrees [3,4]. In different neurodegenerative diseases reduced numbers of proliferating cells were observed in regions of adult neurogenesis (reviewed in [5]), indicating that besides the loss of existing neurons, the adult brain‘s endogenous capacity for cell renewal might be impaired as well. While neurodegeneration is commonly associated with dysfunction of cells, impaired adult neurogenesis is therefore another phenomenon observed in patients with synucleinopathies and models of a-syn related neurodegeneration [5]. This is in particular important since a-syn has a regulatory role in dendritic arborization and spine development in the hippocampal dentate gyrus [6].

In rodents, a number of studies suggest that neurogenesis in the olfactory bulb (OB) contributes to olfactory learning and memory [7,8]. A-syn transgenic (tg) animal models mimic certain neuropathological aspects of synucleinopathies such as PD and have been used to examine the physiological as well as pathological role of a-syn, with respect to adult neurogenesis [9]. Mice expressing WTS under the regulatory control of the human platelet-derived growth factor β (PDGF) promoter accumulate WTS in neurogenic niches resulting in compromised adult neurogenesis in the hippocampal as well as the OB system [10,11].

Tg animals expressing human wild-type a-syn (WTS) under the regulatory control of the murine Thy1 (mThy1) promoter overexpress WTS in a neuron-specific manner and are known to have a particularly high tg expression. Moreover, accumulation of WTS causes progressive, age-dependent neurodegenerative changes with an L-Dopa-reversible sensorimotor phenotype and olfactory dysfunction [10,12–14]. The aim of this study was to investigate the degree and temporal dynamics of adult neurogenesis in these animals.

Compared to PDGF WTS tg mice we found a high expression of a-syn in the olfactory bulb of mThy1 tg mice, but only adult dopaminergic olfactory neurogenesis in mThy1 animals was impaired. Analysis of the temporal expression pattern during stages of adult neurogenesis revealed a later tg expression under mThy1 than PDGF promoter control, while a-syn protein expression is higher in mTHy1 mice. We conclude, that specifically the temporal expression of a-syn during the generation of new neurons, and not only the absolute level of a-syn expression, crucially influences the survival and differentiation of newly generated neurons.

## Materials and Methods

### Animals

**Fig 1:**
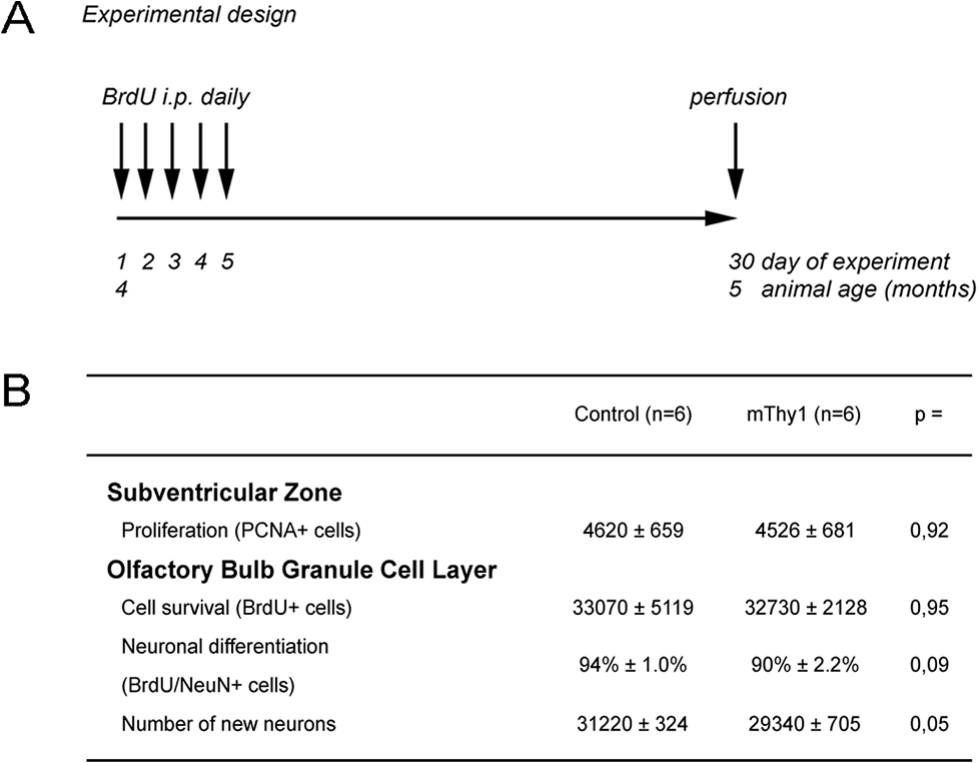
Experimental design. Control and mThy1 a-Synuclein transgenic animals were subjected to a paradigm with 5 consecutive days of i.p. BrdU injections at the age of 4 months and histological analysis at the age of 5 months (panel A). In the SVZ no difference in the number of neuronal progenitor (PCNA-positive) cells was detectable. In the granular cell layer of the olfactory bulb the numbers of surviving BrdU-positive cells, the percentage of BrdU/NeuN double-positive cells as well as the numbers of newly generated neurons were not significantly different in both groups, indicating that mThy1 human wild-type a-Synuclein had no effect on adult granular cell layer neurogenesis (panel B). Numbers are given as means ± standard error means.

All animal procedures were performed in accordance with the protocols approved by the animal care use committee of the University of California, San Diego following National Institutes of Health guidelines for the humane treatment of animals. Animal euthanasia was performed using Xylazin/Ketamin i.p. injections for anaesthesia before transcardial paraformaldehyde perfusion for tissue fixation. All mice were kept in a 12 h light/dark cycle and had access to food and water *ad libitum*. Tg mice expressing WTS under the regulatory control of the mThy1 promoter belonging to the high expression line 61 described earlier [10] were compared to wildtype littermate controls (Ctrl) of the same C57BL6/DBA background (n = 6 per group). To assess the impact of tg expression on olfactory neurogenesis, a standard neurogenesis paradigm for survival of new neurons was applied as described previously [11,15]. Animals received daily intraperitoneal injections with 5-Bromo-2-deoxyuridine (BrdU; 50mg/kg) for 5 days at the age of 4 months and were sacrificed 1 month later (Fig 1A).

### Histology

Animals were perfused transcardially with 0.9% NaCl followed by 4% paraformaldehyde (PFA) in 0.1M phosphate buffer. Brains were removed and stored in 4%PFA and 30% sucrose overnight and then cut sagittally in 40μm frozen sections on a sliding microtome. Sections were stored in cryo-protection solution (ethylene glycol, glycerine, 0.1M phosphate buffer, 1:1:2, pH 7.4) until further processing.

### Primary antibodies and final dilutions

The following primary antibodies were used: rat-α-BrdU (OBT0030, 1:100, Accurate, Westbury, USA), mouse-α-PCNA (P8825, 1:1000, Sigma-Aldrich, St. Louis, USA), goat-α-DCX (sc-8066, 1:250, Santa Cruz Biotechnology, Santa Cruz, USA), rabbit-α-TH (P40101, 1:500, Pel Freez, Rogers, USA), rabbit-α-Sox2 (AB5603, 1:500), mouse-α-NeuN (MAB377, 1:200), mouse-α-TH (MAB318, 1:400), goat-α-Calretinin (AB1550, 1:500, all Chemicon, Temecula, USA), goat-α-GFAP (AB53554, 1:1000, Abcam, Cambridge, UK), and rat-α-human-α-Syn (15G7) 1:10 (Axxora, San Diego, USA), followed by the following secondary antibodies: donkey-α-rat-biotinylated-IgG (712-065-153), donkey-α-mouse-biotinylated-IgG (715-065-151), donkey-α-goat-biotinylated-IgG (705-065-147) (all 1:250, Jackson ImmunoResearch, West Grove, USA), donkey-α-rat-CY3-IgG (712-165-153) 1:200, donkey-α-rabbit-FITC-IgG (711-095-152) 1:200 (both from Jackson ImmunoResearch, West Grove, USA), and donkey-α-mouse-IgG AlexaFluor 555 (A31570) 1:250 (Molecular Probes Inc. Eugene, USA).

### Immunohistochemistry

Free-floating sections were stained as published previously [11]. The incubation in 0.6% H_2_O_2_ in Tris-buffered saline (TBS: 0.15M NaCl, 0.1M Tris–HCl, pH 7.5, 30 min) was followed by blocking steps in TBS/0.25% Triton-X100/3% normal donkey serum for 30 min. Thereafter, the sections were incubated in primary antibody in TBS/donkey serum overnight at 4°C. Sections were then incubated for 1h with biotinylated secondary antibodies according to the primary antibody host. Amplification of signal intensity was achieved using avidin–biotin peroxidase complex for 1h (Vectastain *Elite* ABC kit; Vector Laboratories, Inc., Burlingame, USA) and signal visualization using diaminobenzidin (DAB; 25 mg/ml 0.01% H2O2, 0.04% NiCl in TBS) peroxidase reaction for 10 min.

For the detection of BrdU-labeled nuclei, the following DNA denaturation steps preceded the incubation with primary anti-BrdU antibody: 2 h incubation in 2M HCl at 37°C followed by a pH adjustment step using 10min rinse in 0.1 M boric acid, pH 8.5. All sections were mounted in anatomical order on glass slides and coverslipped with NeoMount (Merck, KGaA, Darmstadt, Germany).

### Immunofluorescence

After the blocking steps in TBS/0.25% Triton-X100/3% normal donkey serum for 30 min the primary antibodies were applied in TBS-donkey serum overnight at 4°C, followed by incubation with secondary fluorochrome-coupled antibodies for 60 min at room temperature and DAPI (D9542, Sigma-Aldrich, St. Louis, USA) counterstaining (1:1000, 5 min) in order to identify cell nuclei. Sections were then dried, mounted and coverslipped with NeoMount. All sections were processed simultaneously under the same conditions.

### Counting procedures

Blinded quantification was achieved using a semiautomatic, brightfield stereology workstation using chromogenic DAB-visualized cell identification (Nikon Eclipse E600 microscope, LEP BioPrecision microscopy table, *Stereo Investigator 7* software, MicroBrightField, USA) as previously described [11].

Every 12th 40μm section (480μm interval) of the left hemisphere was selected from each animal and processed for immunohistochemistry or-fluorescence. The reference volume was determined by tracing the areas using the respective StereoInvestigator software tool. The number of PCNA-positive cells in the SVZ and BrdU-positive cells in the GCL and GLOM of the OB respectively, were counted exhaustively. In the OB GCL, the optical fractionator, a systematic, random counting procedure combining the advantages of an optical dissector and systematic uniform sampling, was used [16]. BrdU-positive nuclei were counted within a 30μm × 30μm counting frame in a 300μm × 300μm counting grid to achieve a Gundersen coefficient of error <0.1. A guard distance of 2.0μm was used during all cell-counting procedures to avoid introduction of errors due to sectioning artefacts. The total counts of positive profiles were multiplied by the ratio of reference volume to sampling volume in order to obtain the estimated number of positive cells for each structure.

To determine the frequency of neuronal and dopaminergic differentiation of newly generated cells, as well as coexpression of a-syn with cell markers of stages of adult neurogenesis, sections were examined using a confocal laser microscope (Nikon Eclipse TE 300 microscope, Plan Fluor 40x objective (Nikon Instruments Inc. Melville, USA), BIORAD Radiance 2700 laser system and *Lasersharp Confocal Assistant* Software (BioRad, Hercules, USA) at a pinhole setting that corresponded to a focal plane of 2μm or less.

Within the anatomical structure of interest, microscopy frames were chosen randomly by the investigator from among every 12^th^ 40μm section as to allow for equal representation of all parts of the structure. To avoid bias, all BrdU positive cells within a confocal frame were analyzed for colocalization. To exclude false positive results, these cells were analyzed using confocal imaging along the full z-axis and sequentially at each fluorophore wavelength to ensure that coexpression of fluorophores was present in identical focal planes. 50 BrdU-positive cells in each region per animal were analyzed for coexpression with NeuN or TH. The percentage of double labeling cells represents the ratio of neuronal or dopaminergic differentiation. Estimated numbers of newly generated cells (BrdU-positive), neurons (BrdU/NeuN double-positive) and dopaminergic neurons (BrdU/TH double-positive) were calculated by multiplication of the number of newly generated cells and the ratio of differentiation.

### Western Blot analysis

For Western blot analysis, cortex and olfactory bulb of mouse brains were homogenized in RIPA Buffer (50mM Tris/HCl pH7.4, 150 mM NaCl, 2 mM EDTA, 1% (v/v) NP-40, 0.1% (w/v) SDS) in the presence of a proteases inhibitor cocktail (Roche) with a douncer homogenizer Potter at 4°C. Protein levels in the tissue homogenates were quantified via BCA protein assay (Thermo Scientific). 80 μg of total proteins were mixed with the same volume of SDS sample buffer (0.125 M Tris/HCl pH 6.8, 4% SDS, 20% glycerol), separated on 15% SDS-PAGE, and blotted onto nitrocellulose membranes (Millipore). The blots were probed with a mouse anti-alpha-synuclein-1 antibody (1:2000, BD Transduction Laboratories) and a rabbit anti GAPDH antibody (1:2000, FL335, Santa Cruz Technology), followed by the incubation with secondary goat anti mouse and goat anti rabbit antibodies coupled to horseradish peroxidase (1:10000 Dianova, Hamburg, Germany). For the detection of proteins, membranes were incubated with the SuperSignal West Pico Sensitivity Substrate (Thermo Scientific) and visualized by VersaDoc gel imaging system (BioRad).

### Statistical analysis

All data are expressed as mean values ± standard error means (SEM). Comparison of the level of neurogenesis with animals of the PDGF strain sacrificed at the age of 4 months was performed based on previously published data generated under identical experimental conditions [11]. Quantified data was normalized to respective wildtype littermate controls. Unpaired, two-sided Student *t*-test or one-way ANOVA followed by a Tukey’s multiple comparison post-hoc test was applied with Prism 4 statistics software (GraphPad, San Diego, USA). The significance level was set at *p* < 0.05 (*), p<0.005 (**) and p<0.0005 (***) as indicated.

## Results

### Proliferation in the SVZ and adult neurogenesis in the OB GCL are unchanged in mThy1 WTS mice

From the proliferative cell pool that resides in the SVZ, newly generated cells divide and migrate along the rostral migratory stream (RMS) to the granule cell layer (GCL) and glomerular cell layer (GLOM) of the OB [17–19]. No significant differences in proliferating cell nuclear antigen (PCNA) positive neuronal progenitor cells in the SVZ were observed between the mThy1 and the control (Ctrl) group (4526 ± 681 vs. 4620 ± 659; p = 0.92). Survival of newly generated 5-Bromo-2´-deoxyuridin (BrdU)-positive cells (Fig 1B) in the OB GCL (32730 ± 2128 vs. 33070 ± 5119; p = 0.95), as well as GCL volume (in mm^3^: 1.09 ± 0.15 vs. 1.16 ± 0.22; p = 0.79) and consecutively the calculated density of BrdU-positive cells (cells/mm^3^: 31250 ± 3401 vs. 29910 ± 4811; p = 0.83) did not differ between mThy1 WTS animals and controls. Also the percentage of BrdU/ neuronal nuclear antigen (NeuN) double positive newly generated neurons (90% ± 2.2% vs. 94% ± 1.0% vs. p = 0.09) did not differ significantly between mThy1 and Ctrl animals. The estimated numbers of newly generated GCL neurons revealed a trend towards a (6%) reduction in the mThy1 group (29340 ± 705 vs. 31220 ± 324; p = 0.05, Fig 1B).

### Reduction of newly generated dopaminergic neurons in the OB GLOM in mThy1 animals

**Fig 2:**
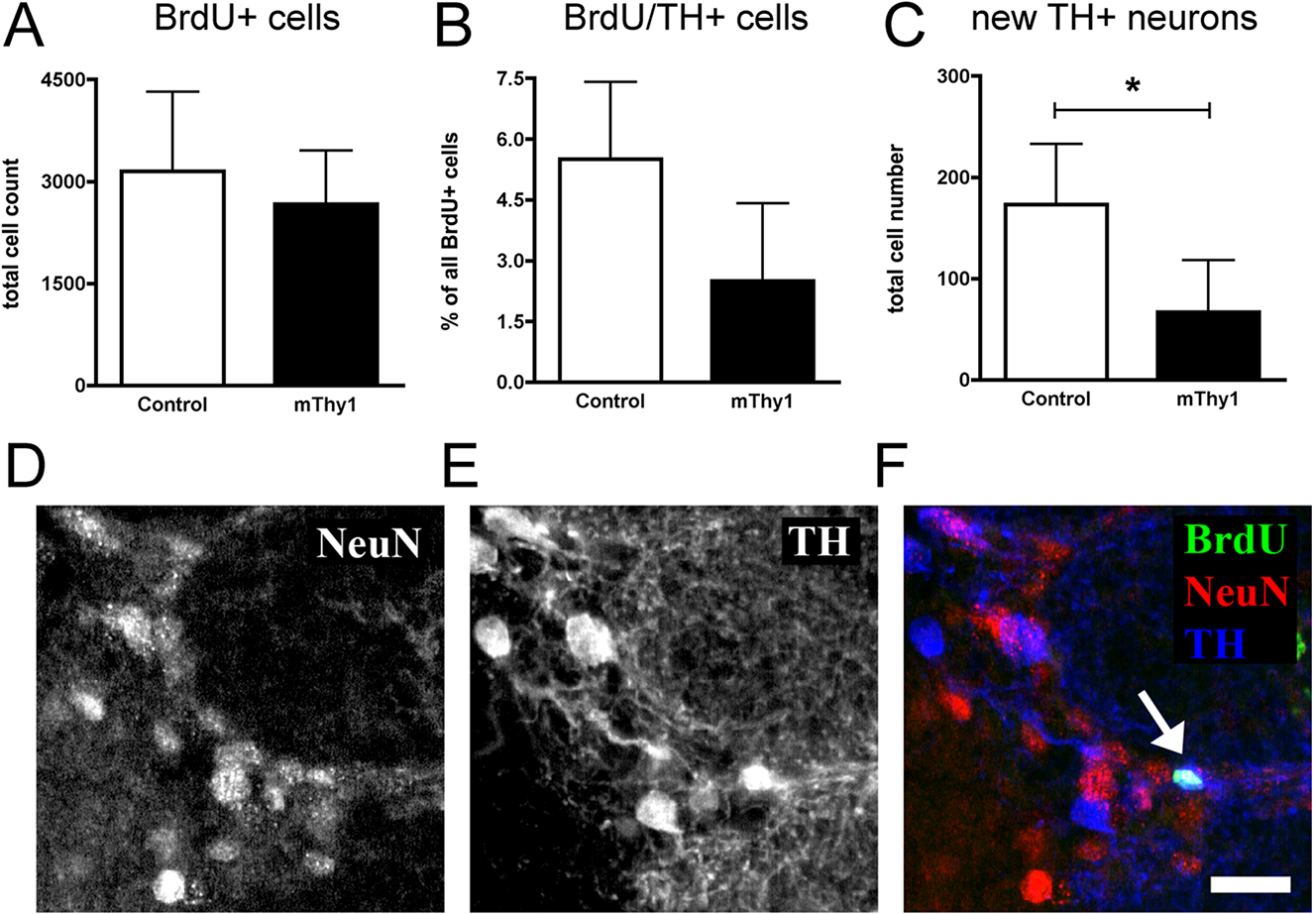
Stereological quantification in the olfactory bulb glomerular cell layer. the number of BrdU-positive cells did not differ between transgenic a-Synuclein mThy1 and control animals (panel A). The analysis of the percentage of BrdU/TH-double-positive cells revealed a non-significant (p = 0.07) trend (panel B). The numbers of newly generated dopaminergic neurons are significantly reduced in the a-Synuclein transgenic animals (p = 0.04, panel C). Immunofluorescent confocal images of the glomerular cell layer show colocalization of markers of neuronal (NeuN, panel D) and dopaminergic differentiation (TH, panel E) with BrdU, a marker of cell survival (panel F). Scale bar 20μm.

The number of BrdU-positive cells in the OB GLOM did not differ between mThy1 animals and controls (2666 ± 394 vs. 3148 ± 586; p = 0.52, Fig 2A). However, the quantification of BrdU/ tyrosine hydroxylase (TH)-double-positive cells as a differentiation measure of newly generated dopaminergic neurons showed a trend towards decreased dopaminergic differentiation in the mThy1 animals (2,5% ± 1.0% vs. 5,5% ± 1,0%; p = 0.07, Fig 2B). Newly generated dopaminergic neurons in the GLOM were significantly reduced in mThy1 animals by 61% (67 ± 26 vs. 173 ± 30; p = 0.04, Fig 2C).

### Comparison to a previously published murine WTS tg model

**Fig 3:**
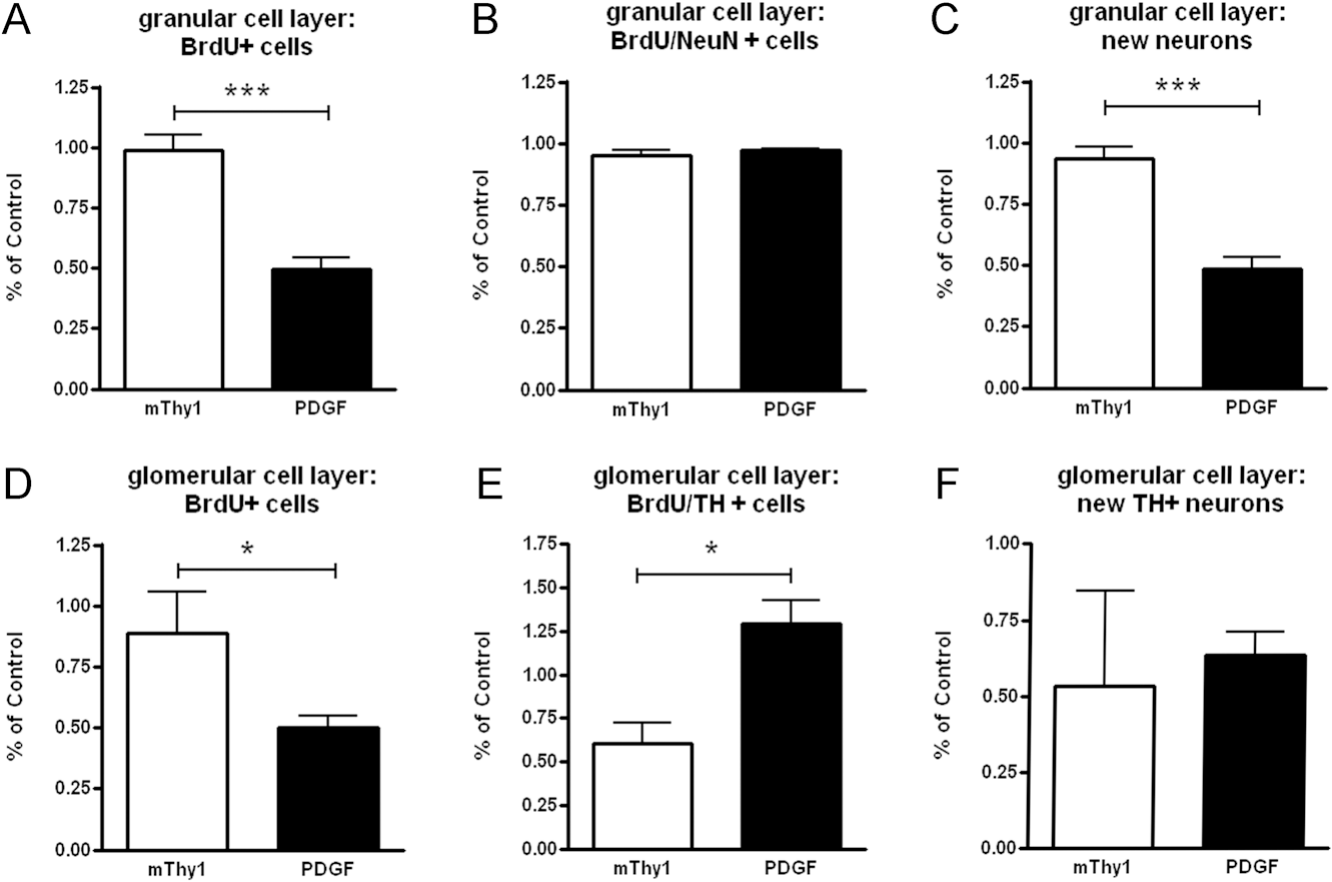
Comparison of olfactory bulb neurogenesis in mThy1 & PDGF transgenic mice. Stereologically quantified numbers of cell survival (BrdU-positive cells: panels A, D), percentage of neuronal / dopaminergic differentiation (BrdU/NeuN+ in panel B, respectively BrdU/TH+ cells in panel E) and new neurons (panels C, F) both in the granular cell layer (panels A, B, C) and the glomerular cell layer (D, E, F) of the olfactory bulb. Results were normalized to the numbers of corresponding control animals of each transgenic animal strain. In the granular cell layer, the number of new neurons was decreased in PDGF animals, while mThy1 animals display an unaltered neurogenesis. In the glomerular cell layer however, there was a reduced cell survival in PDGF, respectively a drop in dopaminergic differentiation in mThy1 animals causing a significant reduction in the number of new dopaminergic cells in comparison to respective control animals, which was not significantly different between both transgenic animal strains.

In PDGF WTS tg mice we previously found a 34% reduction in the number of newly generated GCL neurons, as well as a 59% reduction in newly generated GLOM dopaminergic neurons [11,15]. To compare OB neurogenesis in mThy1 and PDGF WTS animals, we normalized the previously published data [11] and current data with the respective control group of each tg strain and calculated changes in percentage (Fig 3).

Proliferation in the SVZ was unchanged in mThy1 and PDGF animals (0.98 ± 0.15 vs. 0.98 ± 0.07; p = 0.99). In the GCL of the OB, the numbers of BrdU-positive cells and new neurons were significantly different between mThy1 and PDGF animals (0.99 ± 0.06 vs. 0.50 ± 0.04; p = 0.0002; Fig 3A, and 0.93 ± 0.05 vs. 0.48 ± 0.05; p = 0.0002; Fig 3C). In the GLOM of the OB, a significant reduction of BrdU-positive, surviving cells in the PDGF group was observed (mThy1: 0.89 ± 0.17 vs. PDGF: 0.50 ± 0.05; p = 0.02; Fig 3D). Dopaminergic differentiation was significantly reduced in the mThy1 group (mThy1: 0.61 ± 0.12 vs. PDGF: 1.29 ± 0.14; p = 0.01; Fig 3E). Numbers of new dopaminergic neurons were reduced in both groups (0.53 ± 0.31 vs. 0.63 ± 0.08; p = 0.64; Fig 3F).

### WTS expression in mThy1 and PDGF tg mice

**Fig 4:**
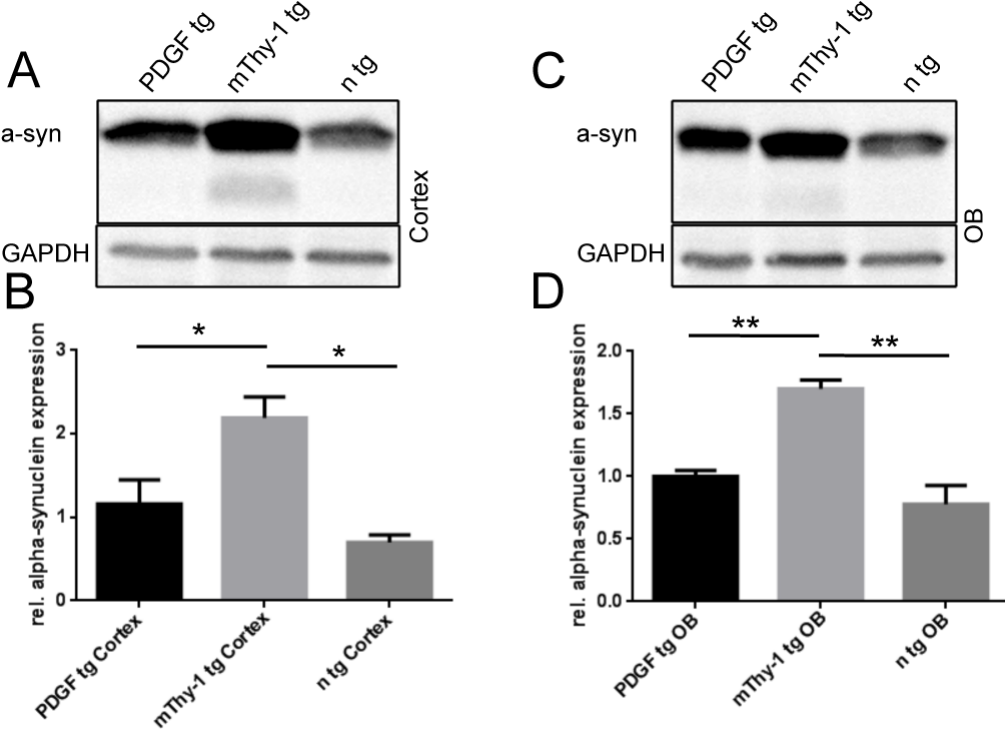
Western blot (WB) analysis of a-Synuclein protein levels. mThy1 and PDGF transgenic a-Synuclein mice and non transgenic littermates (n tg) are compared: (A, C) WB and (B, C) densitometric analysis (normalized against GAPDH) shows a significant increase in a-Synuclein expression in mThy1 mice both in the cortex (A, B, *<0.05, n = 3 replicates, data shown as means ± SEM) and in the olfactory bulb (C, D, *<0.005).

The expression levels of WTS mRNA and protein have been reported to be 4–8x higher in mThy1 than in PDGF animals [10,20]. To specifically compare protein levels of a-syn in the OB, we performed Western blot analysis of cortical and OB areas. We found, that expression of a-syn is significantly higher in mThy1 WTS tg mice than in PDGF WTS tg mice (Fig 4), both in the cortex (Fig 4A and 4B) and in the OB (Fig 4C and 4D). We therefore conclude, that the quantity of tg expression has only a minor impact on modulation of adult OB neurogenesis in this model.

### Temporal expression pattern of WTS in mThy1 tg animals

In PDGF animals it has been shown that during adult neurogenesis in the SVZ/OB system, tg a-syn is expressed from very early stages of cell maturation, as positive colocalization of a-syn with Doublecortin (DCX), a marker of migratory neuroblasts [21], has been shown in both SVZ and RMS [11,15].

**Fig 5:**
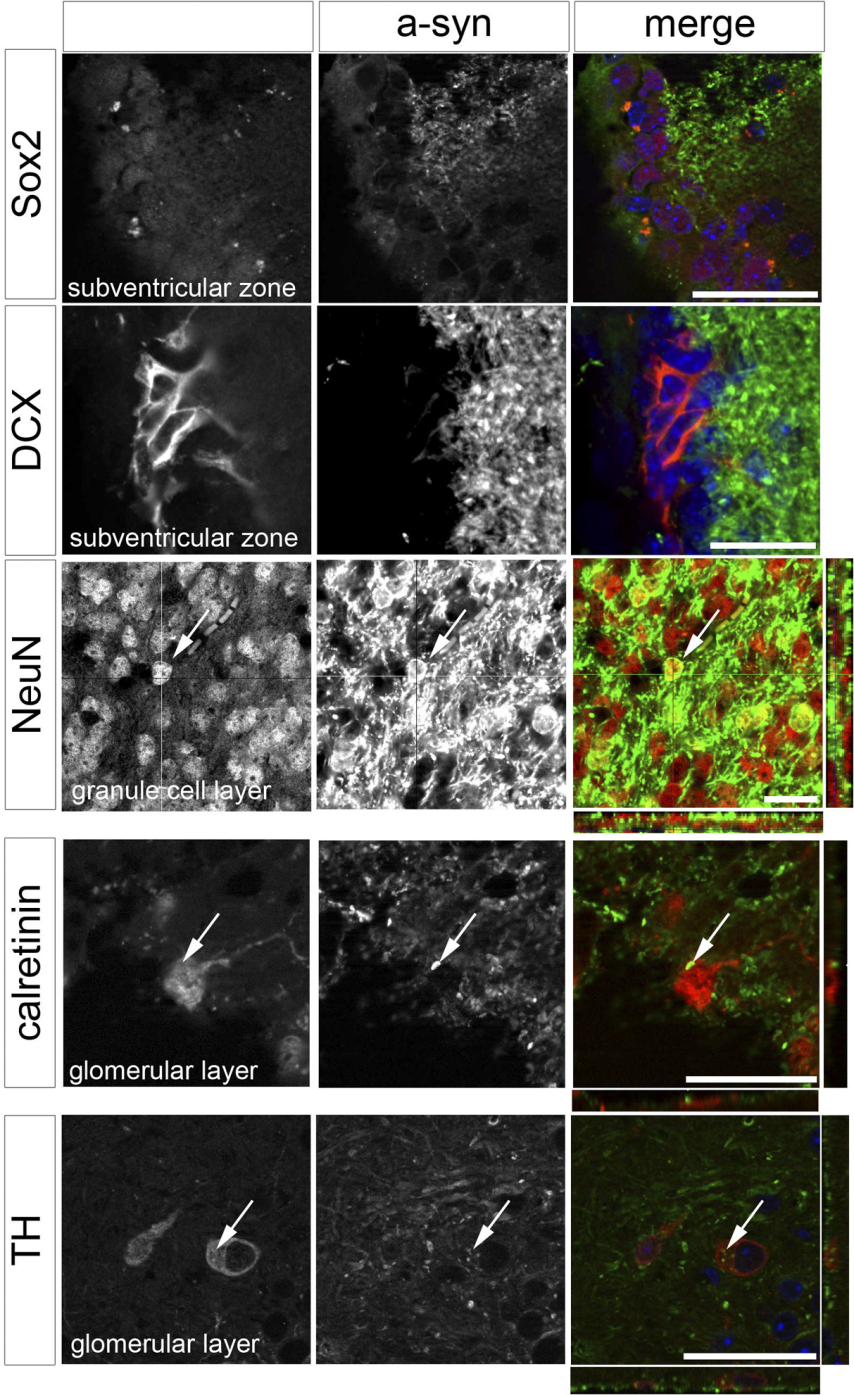
Temporal expression analysis in mThy1 transgenic animals. Coexpression analysis of human-wildtype a-Synuclein with markers of cell maturation from stem cell stage (SOX2), maturing neuroblast (DCX) and mature (Calretinin, NeuN) and dopaminergic neurons (TH). Detailed immunofluorescent analysis in the subventricular zone (Sox2, DCX), respectively olfactory bulb Granule cell layer (NeuN) and Glomerular cell layer (Calretinin, TH) reveals an expression at the mature neuron stage (Calretinin/NeuN-positive). Arrows indicate positive coexpression of human-wildtype a-Synuclein with NeuN, Calretinin and TH. Scale bar 20μm.

To compare the temporal pattern of tg expression during adult olfactory neurogenesis we performed a detailed immunofluorescence coexpression analysis of the tg WTS with established markers of cell maturation in mThy1 animals (Fig 5). For this we used sex-determining region Y-box 2 (SOX2) as a marker of the stem cell stage [17,22], DCX as a marker for migratory neuroblasts, as well as Calretinin, neuronal nuclear antigen (NeuN) and tyrosine hydroxylase (TH) as markers for mature and dopaminergic OB neurons [17].

In mThy1 animals WTS did not colocalize with markers of early stages of adult OB neurogenesis such as SOX2 and DCX, while coexpression was positive in mature neurons. The temporal expression of WTS therefore profoundly differs between the two tg animal strains, such as that WTS is expressed in a much later maturation phase of a newly generated neuron in mThy1 than in PDGF animals.

## Discussion

The neurobiological effect of a-syn on aspects of adult neurogenesis is still not well understood, although in recent years several studies addressed this in both the hippocampal dentate gyrus as well as the SVZ/OB system in tg animal models and murine embryonic stem cells [11,15,23–26]. Specifically the use of conditional tg mouse models has proven that the impairment of adult neurogenesis is reversibly modulated by a-syn [27–29]. In this study we investigated the effect of high levels of WTS expression under the control of the mThy1 promoter on different aspects of OB adult neurogenesis and delineated a dissociation of the expression level versus temporal expression pattern on survival and differentiation of new neurons by comparison to the PDGF WTS tg mouse.

### Impairment of dopaminergic olfactory bulb neurogenesis may be linked to olfactory deficits in mThy1 animals

Adult OB neurogenesis is regulated by external cues such as odor exposure [18,30–32]. It has been convincingly shown that a continuous turnover of newly generated neurons from the SVZ/OB system that integrate as interneurons to the network of the murine OB optimizes olfactory performance [8,18,33]. Our results indicate unchanged rates for SVZ proliferation and survival of newly generated cells in the GCL and GLOM of the OB in mThy1 animals, but a trend towards a reduction in dopaminergic differentiation in the GLOM (p = 0.07) and a significant reduction of newly generated dopaminergic neurons in the GLOM by 61% (p = 0.04).

Repeatedly studies have found a functional connection between dopaminergic OB neurogenesis and olfactory function in the past: In a murine model of impaired neural progenitor cell migration, reduced levels of dopaminergic OB neurogenesis were associated with deficits in fine olfactory discrimination tasks [34]. Furthermore the knock-out of midkine, a growth factor involved in cell migration and neurogenesis, has been shown to cause a partial loss of dopaminergic neurons and depletion of dopamine in the OB, resulting in pronounced olfactory discrimination deficits [35]. A loss of dopaminergic neurons in the OB GLOM by introducing a dopaminergic lesion (6-OHDA into the OB GLOM) caused olfactory deficits that were restored by endogenous adult neurogenesis within one month on a cellular and two months on a functional level [36].

Previous behavioral studies in mThy1 WTS tg animals used paradigms of odor detection, discrimination and identification and showed—among other sensorimotor deficits—a significant olfactory dysfunction, present at the age of 5–6 months [12,37]. At this time point protein-K resistant a-syn inclusions were reported throughout the OB [37]. Our results of decreased levels of OB neurogenesis in this murine model offer an additional path of explanation for this behavioral deficit and confirm previous speculations in this direction by Fleming and colleagues [38].

### Temporal expression of a-Synuclein separately influences cell survival and differentiation in newly generated OB neurons

We found higher levels of WTS expression in the OB of mThy1 than PDGF tg animals, confirming previous studies [10,14,37]. The significant reduction of newly generated dopaminergic neurons in the GLOM of mThy1 animals was due to a reduction in dopaminergic differentiation at unchanged levels of proliferation and survival. In contrast, the marked deficits in adult OB neurogenesis in PDGF animals were mainly due to decreased cell survival (-50%) at an unchanged level of neuronal (GCL) and dopaminergic (GLOM) cell differentiation resulting in decreased numbers of newly generated neurons by 50% in the GCL and 59% in the GLOM [11].

Reports from cell-free experimental systems, yeast, neurons derived from mouse embryonic stem cells, as well as human SCNA-gene duplications and triplications indicate a dose-dependent effect of a-syn toxicity [39–43]. WTS expression in mThy1 brains has previously been shown to be 4–8x higher than in the PDGF strain on an mRNA level [10,20], which we confirmed by demonstrating a significant difference in a-syn protein expression. In comparison to the severely impaired OB neurogenesis found in PDGF animals, the relatively mild impairment of adult olfactory neurogenesis in mThy1 animals shown in this work does not support a dose-dependent a-syn toxicity for adult olfactory neurogenesis.

Our interpretation is that not only the expression level, but in particular the temporal expression of WTS in tg mThy1 and PDGF animals seems to influence aspects of adult neurogenesis. The coexpression analysis in this study showed that WTS tg expression during the maturation process of a newly generated neuron commences at a much later stage under mThy1, than under PDGF promoter control. In addition, data from the phylogenetic activity of both promoter elements point in the same direction: while mThy1 reaches a stable expression from postnatal day 7 onwards [44], PDGF is already prenatally active [45].

There are substantial differences in the temporal expression of WTS in PDFG and Thy-1 promoter mice within the neurogenic niche. We speculate that this difference in the temporal expression of WTS accounts for differences in neuroblast survival and differentiation between mThy1 and PDGF animals: earlier expression of WTS during a crucial phase of maturation has a more pronounced effect on cell survival, whereas later expression has a greater influence on neuronal differentiation (Fig 5). The sequence of changes during cell maturation in adult OB neurogenesis are well-known: during the DCX-positive neuroblast phase of neuronal maturation, migration of cells and dendritic growth are regulated while spine formation and growth follow later, when cells become NeuN-positive [46]. The time course of tg expression might therefore influence certain time-dependent sequences of neuronal maturation such as e.g. cell differentiation or spine formation.

This is also corroborated by findings in human embryonic stem (ES) cell cultures that, when transfected with lenti-viral vectors coding for WTS during late stages of differentiation, have been found to exhibit a decreased rate of dopaminergic differentiation [47]. On the other hand several reports describe that murine ES cell cultures transfected with WTS at an undifferentiated, early stage did not show changes in their dopaminergic differentiation capacity [42,48].

One major difference between the PDGF and the mThy1 WTS mice is a different expression pattern of human alpha-synuclein. While in mThy1 mice (line 61) WTS expression can be found mainly in pyramidal neurons throughout the brain and in highest expression in neocortex, hippocampus, olfactory system, thalamus, substantia nigra and brain stem, in PDGF mice (line D), WTS is mainly expressed in the neuropil and non-pyramidal cells of the neocortex, hippocampus and olfactory system [10].

The PDGF promoter does not lead to a major expression of WTS in the nigral system and dopaminergic neurons, while the ubiquitous tg expression under mThy1 promoter control includes the areas containing TH-expressing cells and fibers like the olfactory bulb, striatum, and substantia nigra. It has been observed in dopaminergic lesion models that nigro-striatal denervation can modulate SVZ cell proliferation and increase dopaminergic neurogenesis in the GLOM of the olfactory bulb [49,50]. While one might speculate that widespread WTS expression in the brain and dopaminergic system might lead to major modulations in adult neurogenesis, our results in the Thy-1 promoter model show that this is not the case at the age of 5 months.

PDGF WTS tg mice have been reported to show glial expression of WTS [10]. Although this is found to a lesser extent in the line used in this study (line D), and the low WTS expression in neurogenic niches, it nevertheless cannot be excluded that glial WTS expression might add to the neurogenesis deficit in the PDGF WTS tg strain.

## Conclusion

**Fig 6:**
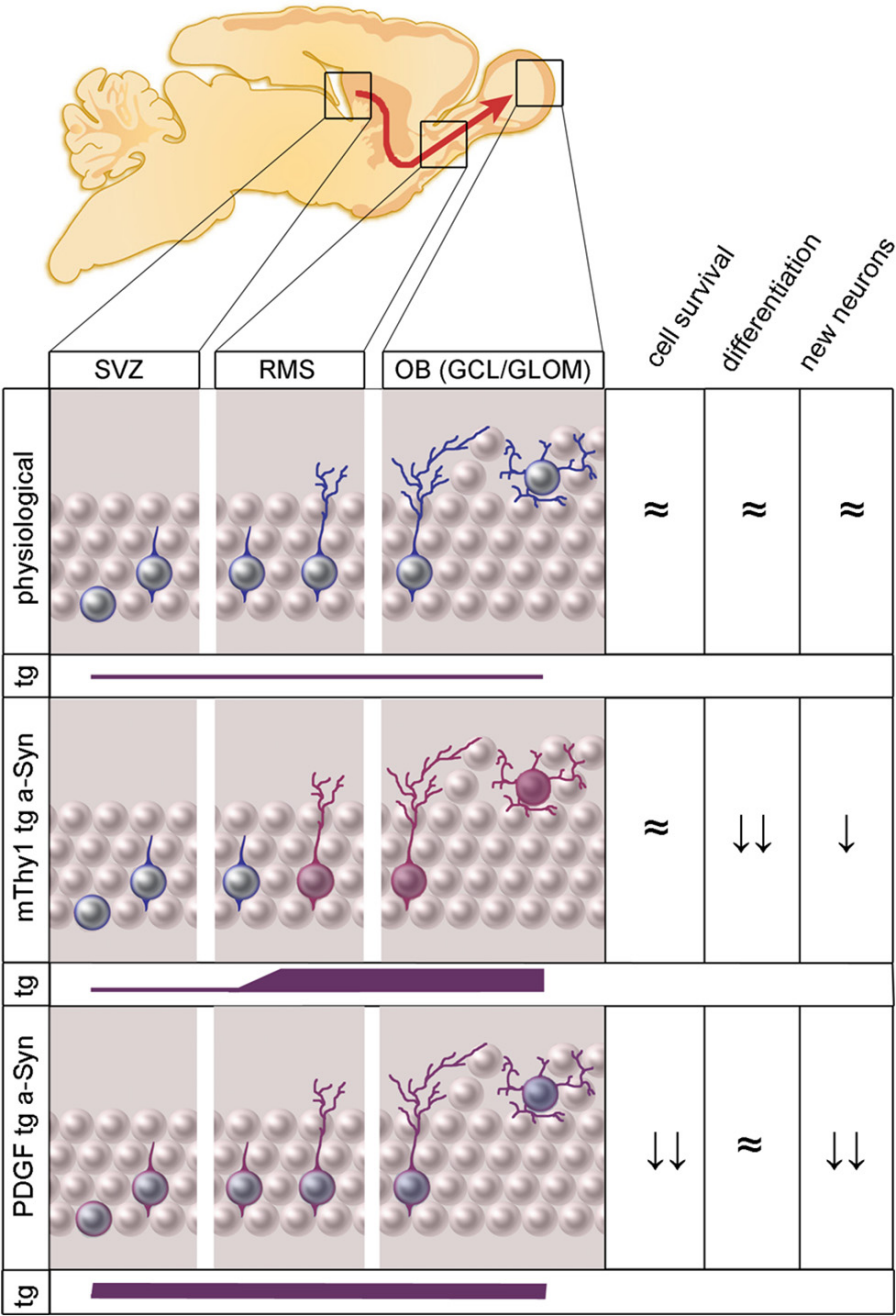
Effect of a-Synuclein expression level and temporal expression pattern on olfactory bulb neurogenesis. The early expression of relatively low levels of transgene (tg, purple bar) in PDGF animals leads to a significant reduction of newborn neurons by affecting cell survival but not cell differentiation. In contrast, a stronger expression of a-Synuclein at later stages of neuronal maturation in mThy1 animals causes lesser changes in adult olfactory neurogenesis mainly affecting dopaminergic differentiation in the GLOM.

In conclusion, this study provides evidence for reduced dopaminergic adult OB neurogenesis in the murine tg mThy1 WTS model due to changed differentiation but not survival of newly generated neurons. This might explain the known olfactory deficits in these mice. Furthermore, the comparison of neurogenesis deficits and tg expression kinetics in different WTS tg mice points beyond a dose-dependent effect of WTS on adult OB neurogenesis. We for the first time show a dissociation of expression levels and their detrimental effect on adult OB neurogenesis in mouse models of synucleinopathies. In the two WTS models compared in this study, the early onset of WTS expression during the maturation of an individual neuron has a stronger effect on levels of adult neurogenesis than a later but much higher tg expression level, providing evidence for the importance of temporal expression characteristics in WTS related experimental neurodegeneration (Fig 6).

This finding may not only help to understand results of adult neurogenesis studies due to promoter differences, but also expands the dimensions of expression characteristics to be studied in future tg models of synucleinopathies—along cell-specificity, anatomy and dose, also temporal tg expression characteristics should be addressed.
